# Variation in breeding practices and geographic isolation drive subpopulation differentiation, contributing to the loss of genetic diversity within dog breed lineages

**DOI:** 10.1186/s40575-020-00085-9

**Published:** 2020-06-09

**Authors:** Sara Lampi, Jonas Donner, Heidi Anderson, Jaakko Pohjoismäki

**Affiliations:** 1 Department of Environmental and Biological Sciences, University of Eastern Finland, P.O. Box 111, 80101 Joensuu, Finland; 2 Wisdom Health, P.O. Box 1040, 00251 Helsinki, Finland

**Keywords:** Genetic differentiation, Heterozygosity, Geography, Conservation genetics, English greyhound, Labrador retriever, Italian greyhound, Shetland sheepdog, Belgian shepherd, Finnish Lapphund

## Abstract

**Background:**

Discrete breed ideals are not restricted to delimiting dog breeds from another, but also are key drivers of subpopulation differentiation. As genetic differentiation due to population fragmentation results in increased rates of inbreeding and loss of genetic diversity, detecting and alleviating the reasons of population fragmentation can provide effective tools for the maintenance of healthy dog breeds.

**Results:**

Using a genome-wide SNP array, we detected genetic differentiation to subpopulations in six breeds, Belgian Shepherd, English Greyhound, Finnish Lapphund, Italian Greyhound, Labrador Retriever and Shetland Sheepdog, either due to geographical isolation or as a result of differential breeding strategies. The subpopulation differentiation was strongest in show dog lineages.

**Conclusions:**

Besides geographical differentiation caused by founder effect and lack of gene flow, selection on champion looks or restricted pedigrees is a strong driver of population fragmentation. Artificial barriers for gene flow between the different subpopulations should be recognized, their necessity evaluated critically and perhaps abolished in order to maintain genetic diversity within a breed. Subpopulation differentiation might also result in false positive signals in genome-wide association studies of different traits.

**Lay summary:**

Purebred dogs are, by definition, reproductively isolated from other breeds. However, similar isolation can also occur within a breed due to conflicting breeder ideals and geographic distances between the dog populations. We show here that both of these examples can contribute to breed division, with subsequent loss of genetic variation in the resulting breed lineages. Breeders should avoid creating unnecessary boundaries between breed lineages and facilitate the exchange of dogs between countries.

## Background

Domestic dogs (*Canis familiaris* L.) are the oldest domesticated animals, comprising of more than 400 highly diverse contemporary breeds [1]. The domestication process itself has caused genetic bottlenecks shaping dog development, the first dated to have occurred about 15,000 years ago [1, 2]. While early domestication was characterized by adaptation to the mutualistic relationship with humans, different dietary conditions and geographical differentiation, the recent bottlenecks are much more dramatic and caused by goal-directed breeding, especially during the last two centuries. In fact, the idea of discrete, uniform and standardized dog *breeds* originates from the Victorian era and current breeding practices largely still reflect this thinking [3]. Creation of breeds was driven by the advent of dog shows, where breed standards are contested and the best dogs in each class rewarded. Competition brings prestige and some income from sales and stud fees for the owners, but more importantly forces the specification of ideal breed conformation.

Establishment of a breed, by definition, causes effective cessation of gene flow to the population from other dog populations. Furthermore, extreme breed standardization as the main driver of breeding practices causes strong reduction of the effective population size and high levels of inbreeding within the breed, resulting in loss of genetic diversity and accumulation of deleterious alleles in many of the contemporary breeds [1, 4–7]. While extreme directional breeding often results in exaggerated morphological or functional characteristics [8], also less specialized breeds – or so-called primitive breeds – are impacted by extreme breeding practices. We have recently shown that the excessive use of champion males has driven the loss of heterozygosity in the Finnish Spitz, in contrast to the Nordic Spitz [9], which is a younger breed with more loosely defined standards. This is particularly interesting as the two breeds originate from the same feral founder population [10], making it possible to compare the outcomes of the different breeding practices in a similar genetic background. It should be noted that breed standardization per se is not a problem – there are many very healthy breeds and also mixed breed dogs can have health problems. Maintenance of healthy breeds requires positive selection for healthy traits, which can be controlled and facilitated by compulsory veterinary checks as well as professional breeding advisory.

Differing breeder preferences and breed ideals can cause additional reproductive isolation within breeds. For example, in gun dogs, selection on fur types and local breed sub types has often resulted in splitting of the breeds [11], but the extent of subpopulation differentiation within a breed due to diverse breeding practices is less known. In many of the contemporary breeds, there are existing divisions depending on the breeding goals as well as geography. If these divisions result in subpopulation differentiation, they could exacerbate the loss of genetic diversity within the breed. In extreme cases, all individuals of the breed might be either affected or carriers of a deleterious trait, making it difficult to eradicate by traditional breeding. For example, almost all individuals in brachycephalic breeds are homozygous for a *DVL2* mutation, whose phenotype could be considered as a developmental defect, causing reduced life quality in these animals [12]. Loss of genetic diversity could also be an underlying reason behind breeding difficulties, reduced life span and compromised immunity [13]. In these cases, genetic rescue by crossbreeding with related breeds might be inevitable [14].

In the presented study, we sought to see whether the differential breeding practices, such as partition to sport or show dogs, or geographical division has caused genetic differentiation into recognizable subpopulations in six popular dog breeds, Belgian Shepherd, English Greyhound, Italian Greyhound, Finnish Lapphund, Labrador Retriever and the Shetland Sheepdog. Using a genome-wide survey of 1319 single nucleotide polymorphisms (SNPs), we show here that all of the studied breeds show subpopulation differentiation, either by their geographical origin, selection for performance or morphological traits. We conclude that the subpopulation differentiation should be taken into account in the breeding programs of the studied breeds to conserve their genetic diversity.

## Results

We analyzed the genetic differentiation in Belgian Shepherd, English Greyhound, Italian Greyhound, Finnish Lapphund, Labrador Retriever and Shetland Sheepdog using multi-dimensional scaling (MDS) [15] and STRUCTURE [16] analysis of the genotypes of the 1319 SNPs. The observed population structure was matched with the ad hoc data on geographical location, use (sport vs. show dog) and morphological traits of the individual dogs (see Methods). The example breeds were chosen as they represent either sport dogs used for competition (Greyhounds), are known to have subpopulations used for service or as companion dogs (Labrador Retriever), have differentially bred sub-types (Belgian Shepherd), are locally (Finnish Lapphund) or globally (Shetland Sheepdog) popular companion dogs.

**Fig. 1:**
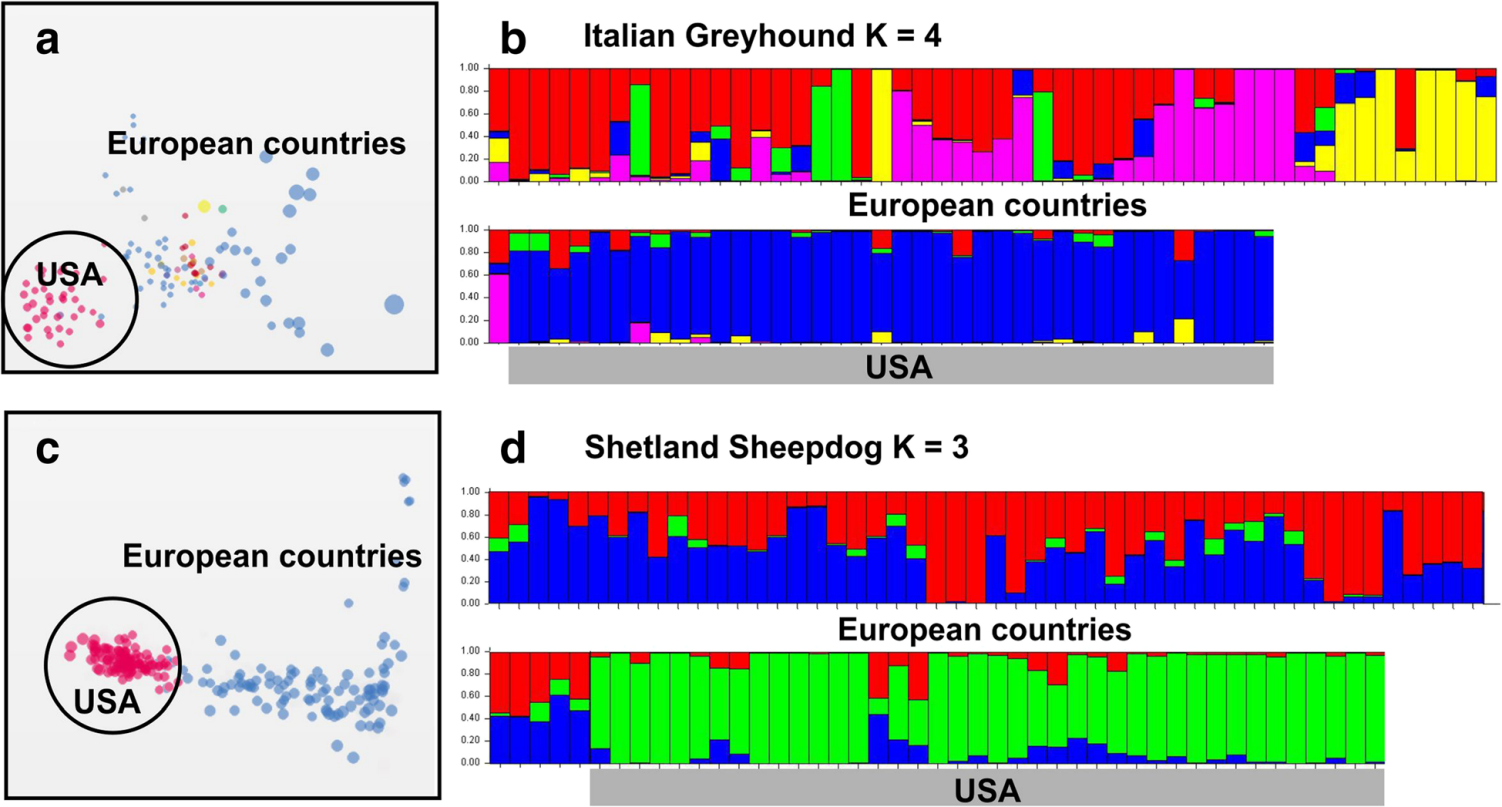
Geographical differentiation in Italian Greyhound and Shetland Sheepdog. **a** The Italian Greyhound samples from the US cluster closely together in the MDS analysis compared to the European samples. Dot colors indicate different countries of origin. **b** STRUCTURE and STRUCTURE HARVESTER analysis revealed four genetic clusters (*K* = 4) within the Italian Greyhound. Each bar in the graph corresponds to an individual and the genetic clusters are represented with different colors. Note the genetic uniformity among the US samples. **c** The Shetland Sheepdog samples show similar pattern as the Italian Greyhound, with the US samples forming a discrete cluster and the European samples being more scattered. Colors as in (**a**). **d** The Shetland Sheepdog samples analyzed in the study belong to three differing genetic clusters. As in (**b**), the US samples are genetically more uniform than the European samples

In contrast to our previous study on Nordic hunting dogs [10], all of the breeds studied here showed at least some level of subpopulation differentiation. Italian Greyhound and the Shetland Sheepdog show clear geographical subdivision with the dogs from different continents clustering together (Fig. 1). It is noteworthy that although STRUCTURE analysis supported ancestral population differentiation among the European dogs in both breeds, as indicated by the different colours (Fig. 1b and d), there seems to be relatively frequent admixture reflecting the exchange and import of breeding dogs between the European countries. The fact that the US populations of both breeds are strikingly different from their European counterparts is likely to be mainly due to a founder effect, resulting in higher *F*^*ST*^ and lower heterozygosity (*Hz*) values (Table 1). Both the Italian Greyhound and the Shetland Sheepdog are European breeds and the US populations originate from probably a handful of imported dogs. While the Italian Greyhounds in the US represent rather uniform population (Fig. 1b), the Shetland Sheepdog seems to have two dominant ancestral populations (Fig. 1d).

**Table 1:** The degree of heterozygosity (*Hz*) and subpopulation differentiation (*F*^*ST*^) in the six studied breeds

	Median ***Hz***	***F***^***ST***^
**Italian Greyhound**		
European population	0.3466	0.0846
US population	0.3199	0.1548
**Shetland Sheepdog**		
European population	0.3029	0.1847
US population	0.289	0.2266
**English Greyhound**		
Dog sports	0.338	0.0665
Show / Companion	0.2606	0.3405
**Labrador Retriever**		
Working dogs	0.3934	0.0071
Show / Companion	0.3372	0.1858
**Belgian Shepherd**		
Groenendael	0.3485	0.198
Malinois	0.3938	0.0881
Tervueren	0.3768	0.1869
Laekenois	0.3211	NA^a^
**Finnish Lapphund**		
Herding background	0.3724	0.1182
Other	0.399	0.0343

^a^*F*^*ST*^ for Laekenois was not available as the sampled dogs representing this breed variety were not distinguishable from Malinois

**Fig. 2:**
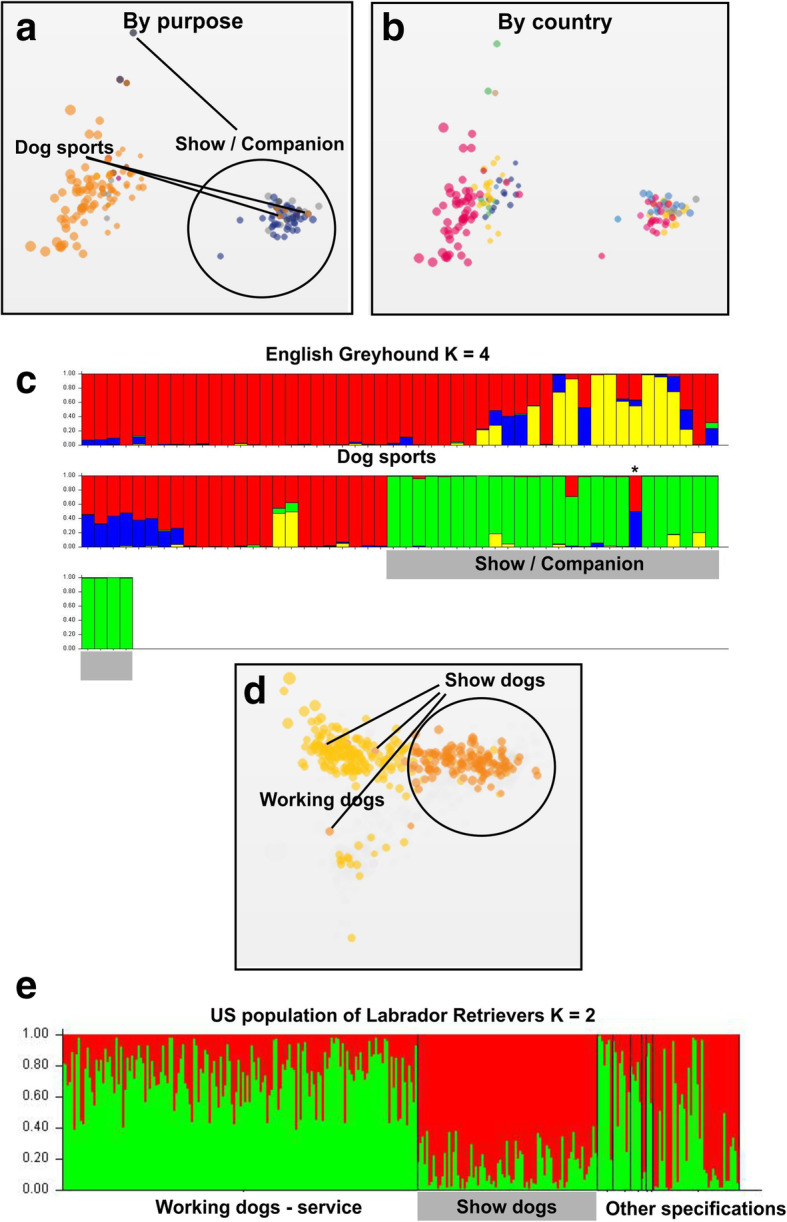
Lineage purpose differentiation in English Greyhound and Labrador Retriever. **a** The English Greyhound sample clusters correlate with their stated purpose as given by the dog owner, but not with the geographical location (**b**). Dot colors in (**a**) according to sport and show or companion status and in (**b**) according to the country of origin. As the use of the dog for a purpose is not biologically determined, there are several examples (marked with lines) where the use does not correlate with the genotype. **c** As with the Italian Greyhound, the English Greyhounds contain four genetic clusters. Concomitant with the MDS, the show or companion dogs form a more uniform cluster than the racing dogs. The asterisk (*) points to an outlier individual, which genetically belongs to the racing dogs but has been tagged as a companion dog. **d** Similarly, the show lineage Labrador Retrievers form a tight cluster compared to the working dogs. For simplicity, only the US originating dogs were included in the analyses. The working dogs include various assistant and service dogs as well as dogs used for hunting. These did not, however, cluster by the specific type of use (**e**)

In the English Greyhound and in the Labrador Retriever, selection for performance, either in dog sports or as service dogs, explained the genetic subpopulation differentiation (Fig. 2). The majority of the English Greyhounds reported by their owner to be show or companion dogs clustered together both in MDS (Fig. 2a) as well as in STRUCTURE analysis (Fig. 2c), without showing much correlation with the country of origin (Fig. 2b). Labrador Retrievers showed both strong geographical differentiation and lineage differentiation. The lineage differentiation was more evident when the analysis was focused only on the US population. The differentiation of the American Labrador Retrievers into show and working/service dog lineages was evident both in MDS (Fig. 2d) as well as in STRUCTURE (Fig. 2e). Interestingly, in both cases the show dog lineages were more differentiated i.e. having higher *F*^*ST*^ values than the working dog populations (Table 1).

**Fig. 3:**
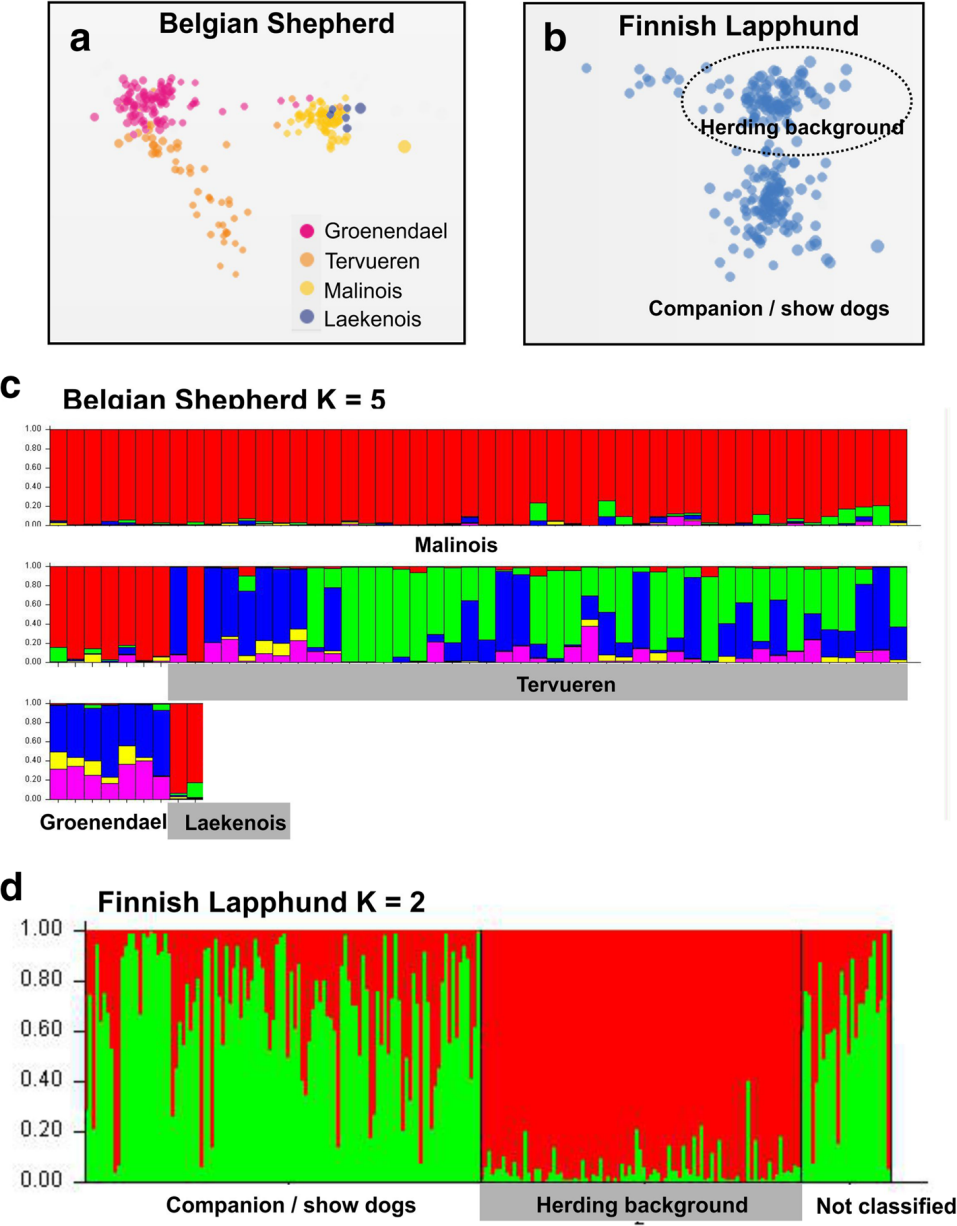
Breed type differentiation in Belgian Shepherd and Finnish Lapphund. Both the Belgian Shepherd (**a**) as well as the Finnish Lapphund (**b**) samples form discrete clusters in the MDS analysis. While there are established breed forms of the Belgian Shepherd, which – with some inconsistencies (see the Discussions) – correspond to the genetic clusters discovered in the STRUCTURE analysis (**c**), the clustering of the Finnish Lapphund samples (**d**) correlate with the pedigree lineages preferred by breeders. Note that the most of the Lapphund, regardless of their pedigree, are used as companion dogs and the subpopulation names in the figure do not necessarily reflect their contemporary usage. The “herding background dogs” are descendants of original Sámi working dogs, whose breeding follows the rules set by their corresponding breed association

The Belgian Shepherd Dog has four different varieties: shorthaired Malinois, wirehaired Laekenois, and longhaired Tervueren and Groenendael. Tervueren and Groenendael differ by their coat colour, the former being sable and the latter black. In the US, the name “Belgian Sheepdog” is reserved for and used when referring to Groenendaels. The US originating “Belgian Sheepdogs” were omitted from the analysis as, although clustering with the European Groenendael, they also showed population differentiation caused by founder effect. As expected, the Malinois population consisting mostly of working dogs, forms its own distinct cluster (Fig. 3a). Rather unexpectedly, this cluster also contains the sampled Laekenois; a finding also supported by the CLUSTER analysis (Fig. 3c). Despite their coat type difference, both breed forms are used as working dogs and crossbreeding of the two types is allowed, at least in Finland, where the Laekenois specimens originated from. The longhaired Groenendael and Tervueren were more heterogenic, with some Tervuerens being indistinguishable from Groenendaels.

The last breed in our analysis was the Lapphund, a fairly young breed, originating from the Sámi reindeer herding dogs together with the Lapponian herder [10]. In contrast to the other primitive spitz-type Nordic breeds, including its sister breed the Lapponian herder [10], the Lapphund shows distinct subpopulation differentiation (Fig. 3b and d). Based on our manual analysis of the pedigrees of the sampled dogs, the cause of this differentiation can be traced to the breeding practises of a Finnish Lapphund breeding association. The goal of the association has been to maintain traditional reindeer herding dog type and therefore has strongly restricted the use of sires outside of the lineages approved by the association. This restrictive breeding is evident also in the CLUSTER and *F*^*ST*^ analysis (Fig. 3d, Table 1), showing gene flow from the Finnish Lapphund population of herding dog descendants into the conventional show dog population but not vice versa. The speed of the differentiation is remarkable, as the association following the restricted breeding practises was founded in 1981.

## Discussion

In general, the efficacy of population genetic studies is dependent on the sample size as well as on the number and type of the chosen markers. Although dwarfed by the existing 170 k arrays for dogs [6, 17, 18], the 1319 diagnostic neutral SNPs used in the study have previously been successfully used in detecting dog population structure as well as inferring their genetic relationships [9, 10]. In more detail, as the distance between the markers is known, the 1319 SNPs were successfully used for an *r*^*2*^ based estimation of the historical effective population sizes of the Finnish and Nordic Spitz [9]. In general, analysis of SNPs across all chromosomes can outperform any traditionally used STR panels in population genetic analyses [19]. When several loci are used, as in the case of whole-genome analyses, sample sizes of 20–30 individuals are considered to give a sufficient overview of the studied population [20, 21]. Our sample sizes varied from 90 to 608 (Table 2) and even the smaller sample set did not show any singleton genotypes (Figs. 1, 2 and 3). The same genotyping panel is also used by the dog owners to test if their dogs are carriers of known single-locus Mendelian disorders, or other functional variants, but these loci were not included in our analyses. While certain disease variants might be enriched in some breed subpopulations, these do not influence the genetic makeup of the breed. Breeders also often test several related dogs (parents, offspring, siblings), which could have influence in the representation of individuals within each *K* when the small sample size is small. However, as the related dogs represent the same geographical region and breed type, this type of biases would not affect the identification of subpopulation divisions.

**Table 2:** Breeds, sample sizes and the hypothetical population division used in the study

Breed	Sample size	Hypothetical population based on
Belgian Shepherd	142	Breed form
English Greyhound	104	Country of origin or lineage purpose
Finnish Lapphund	224	Breeding restrictions
Italian Greyhound	90	Country of origin or lineage purpose
Labrador Retriever	608	Country of origin or lineage purpose
Shetland Sheepdog	95	Country of origin

Our analysis was able to demonstrate subpopulation differentiation in all of the studied six breeds and confirmed that both geographical isolation as well as differential breeding strategies can have similar outcomes and that the differentiation can be fairly rapid, as seen in the Finnish Lapphund. If the reproductive isolation was to be complemented with strong directional selection, we would expect the differentiation to be even faster and more extreme. It is noteworthy that the differentiated subpopulations having high *F*^*ST*^ values (Table 1), formed tight clusters on the MDS plots and were more uniform in their STRUCTURE charts than the less differentiated examples (Figs. 1, 2 and 3).

The geographic differentiation between the European and the US populations of the Italian Greyhounds and the Shetland Sheepdogs was expected. Both breeds originate from Europe and the US populations are based on few founders [22]. The founder effect combined with the limited gene flow between the continents is expected to be efficient drivers of subpopulation differentiation. What is more astounding is that similar or even more extreme differentiation rates, as evident in the *F*^*ST*^ values (Table 1), were observed in subpopulation division to sport/working dogs and show dogs in English Greyhounds and Labrador Retrievers. In addition, one might expect that selection for running performance or work, respectively, would be a dominant driver in the differentiation, but in both breeds the show dog subpopulations had higher *F*^*ST*^ values. This probably reflects the overrepresentation of few champion breeders and hence low effective population size in the show dog lineages. There also seems to be gene flow from the show lineages to the sport or working dogs but not vice versa, also reflecting differential breeding practices in the corresponding kennel cultures.

The Belgian Shepherd is an interesting example of a dog breed with several different breed varieties with discrete breeding practices. Although the separation into breed varieties has driven subpopulation differentiation, the fact that the sub types are determined by coat type and color, both monogenic traits, can result in a situation where genotypically similar individuals are considered different breed types by the breed community (Fig. 3c). For example, the lack of differentiation between Groenendaels and Tervuerens can be explained by the fact that the former exhibit the dominant black in coat colour, but can produce sable-coated offspring, which are registered as Tervuerens. Registration of offspring under a breed variety different to that of its parents plays a role in allowing gene flow between the subpopulations.

In contrast to the Belgian Shepherds, there are no phenotypically separated breed types in the Finnish Lapphund. Instead, a breeder association preferring certain family lineages to others and setting restrictions on outbreeding with the remaining population likely drives the subpopulation differentiation in this breed. The goal of this selective breeding has been to protect the original identity of these reindeer herding dogs; however, as all animal populations evolve over time, it is uncertain how this goal can be objectively evaluated. In fact, the original Sámi reindeer herding dogs have been artificially split into two breeds based on their coat type, the Lapponian Herder and the Finnish Lapphund. Finnish Lapphund is among the most popular breeds in Finland, with some 1224 registered in 2019 and only a tiny fraction of these dogs are in any herding use.

Population fragmentation is an issue for the conservation of the genetic diversity in any population. The measure of population differentiation *F*^*ST*^, by definition, is in effect a measure of inbreeding in the subpopulation relative to the total population [23, 24]. Inbreeding itself reduces the heterozygosity in a population and in fact inbreeding can be expressed also as a function of loss of *Hz* over generations (see [9] for the different metrics and discussion in dogs). This is also evident in our study, where the subpopulations with the highest *F*^*ST*^ had also the lowest *Hz* (Table 1). If the obstacle for the gene flow is maintained, *Hz* will continue to erode at higher rates than in the less differentiated populations. Two factors influence the *F*^*ST*^ and *Hz*. First is the effective population size *N*_*e*_, which is not simply the number of individuals contributing to the next generation, but is also dependent on the relatedness of these individuals, affecting the subsequent change in the inbreeding rate of the population (see Kumpulainen et al. for discussion regarding dogs [9]). In essence, pairing closely related dogs, as often the case for line breeding, results in higher inbreeding rate and faster loss of *Hz* over generations. The *N*_*e*_ can dramatically reduced, at least temporarily, due to founder effect when a dog breed is introduced to a new country. The founder effect was also evident in our study, where certain European ancestral lineages were strongly enriched and others absent in the representatives of the American lineages (Fig. 1b, d). Similarly, much of the DLA variation has been lost due to founder effect in the US populations of several European dog breeds [25], which could have functional consequences for their immunocompetence. For example, the US population of Italian Greyhounds, also included in this study, was found susceptible for many autoimmune diseases not observed in the European population [22]. Secondly, as the drift operates in each generation, the longer the subpopulation is separated from the others, the larger the *F*^*ST*^ will be.

The loss of genetic diversity can pose a threat to breed health [13, 22, 26–29]. Although this loss cannot be completely avoided due to the closed population nature of all dog breeds, some breeding practices, such as extreme selection for “best in breed” winners of competitions and trials or lineages with culturally valued pedigrees have the potential to accelerate the process. We also acknowledge that breed differentiation can serve a purpose, such as the maintenance of working lines, in which case the *F*^*ST*^ can be seen as a measure of an increase in the “quality” genes. In these cases, mixture of differentiated subpopulations could create an inferior outcome, similar to the decay of locally adapted allelic combinations known to occur in the wild, also known as outbreeding depression [30] or migration load [31]. However, contrary to the locally adapted wild populations, dog breeds result from man-made criteria, whose justification should be critically evaluated and diverse breeding options preferred over lineage selection. Also the loss of genetic diversity does not automatically have adverse effects, especially if there is simultaneous positive selection on health, facilitated by rigorous veterinary checks. The health checks, including genetic testing of disease carriers, can also narrow down the breeding population, contributing to the loss of genetic diversity especially in small breeds.

Knowledge of the genetic differentiation of populations and gene flow between them not only offers insights into breed history and current breeding practices, but also highlights how subpopulations might most beneficially be used for the maintenance or restoration of genetic diversity. Optimally the breeding of desired traits should aim to mimic the natural selection operating in wild populations, selecting uniformity for some parts of the genome, while maintaining diversity elsewhere [32, 33]. The simplest tools here might be to maintain large effective population size and constrain inbreeding, which most animals tend to avoid under natural conditions [34]. We also expect that subpopulation differentiation can result in false positive (or negative) signals in genome-wide association studies of different traits and should therefore be taken into account when designing such studies.

## Conclusions

Our results show that geographical isolation and founder effect are the main drivers of subpopulation differentiation among Italian Greyhounds and Shetland Sheepdogs, whereas selection for show and sports or working lineages explained the genetic structure among English Greyhounds and Labrador Retrievers. The genotyping analysis could also detect the predicted breed type structure in Belgian Shepherds and revealed unexpected, relatively recent split in the Finnish Lapphund population due to breeder preferences. Our study exemplifies the use of genetic data in detecting population structure among dogs to identify and measure the degree of subpopulation differentiation. The findings could be then applied in breeding programs to facilitate gene flow between isolated subpopulations, as well as to raise awareness of practices that are potentially harmful for the maintenance of genetic diversity within a breed. Responsible dog breeding requires careful balancing between the selection for the desired traits and health, while avoiding excess loss of genetic diversity due to drift.

## Methods

### Breed selection and DNA sampling

The Belgian Shepherd, English Greyhound, Italian Greyhound, Labrador Retriever, Finnish Lapphund and Shetland Sheepdog breeds were selected for the study as they were known or suspected to have either distinct breed varieties (Belgian Shepherd) or differential breeding practices depending on the intended use of the dogs (Table 2). Breeds without expected subpopulations were not included in this study, as we have recently analysed several Nordic hunting dog breeds using the same methods as a proof-of-concept, and shown that these breeds exhibited no subpopulation division despite evidence of ancestral admixture [10].

Dogs included in the study were sampled as a part of submission for routine commercial screening provided by Genoscoper Laboratories Oy, Helsinki, Finland (MyDogDNA™ or Optimal Selection analysis™ analysis). Additional information on country of origin, breed variety, and owner-reported characterization of the dog’s use (e.g., companion dog, show dog, working dog, dog sports) was collected. The pedigree background of the Finnish Lapphund samples was validated manually based on publicly available breed registry information, assigning the dogs into subpopulations depending on whether their breeder adhered to the herding lineage requirements set forth by the breed community or not.

Genetic analyses were carried out on DNA extracted from owner-collected, non-invasive buccal swab samples. All dog owners provided consent for the use of their dog’s DNA sample for research purposes.

### Genotyping

The genotypes were obtained using a custom-designed Illumina Infinium (Illumina, Inc., San Diego, CA, USA.) genotyping microarray for 1319 neutral SNPs, evenly distributed across the 39 canine chromosome pairs [35] with a median intermarker distance of 1585 kilobases. The array was previously shown to reliably differentiate between breeds, detect population structure and trace their ancestry [10]. The spacing of the markers allows also the estimation of the effective population sizes based on the decay of linkage disequilibrium [9]. Genotypes for all samples are provided as Additional File 1.

### Genetic and statistical analyses

Samples included in this study reached a call rate of at least 95% of the analyzed markers. The median heterozygosities, basic population genetic parameters and *F*^*ST*^ values were calculated based on all individuals using ARLEQUIN v.3.5 [36]. The genetic differences between individuals were illustrated using multidimensional scaling (MDS) analysis [15], which is an eigen-decomposition principal component analysis transforming distances into similarities. The original, three dimensional, rotatable MDS plots are available for all breeds at https://mydogdna.com/ by searching for the breed of interest and choosing the “genetic relationships” tab. The population structures were analyzed using STRUCTURE v.2.3.4. with 5000 MCMC repetitions and burn-in period of 5000 [16, 37]. The most probable subpopulation number was computed using the Evanno method as provided in STRUCTURE HARVESTER [38]. The obtained population structure was then compared against the hypothetical population division based the metadata on the breed form, country of origin or use of the dog.

## Supplementary information

**Additional file 1.**

## Data Availability

All relevant data are provided with the paper and its Supporting Information files.

## Abbreviations

*F*^*ST*^: Inbreeding due to subpopulation differentiation
*Hz*: Heterozygosity
MCMC: Markov chain Monte Carlo
MDS: Multidimensional scaling
*r*^*2*^: A measure of linkage disequilibrium between biallelic markers or same as the square of the correlation coefficient between the presence or absence of a particular allele at the first locus vs the second locus
SNP: Single nucleotide polymorphism
STR: Short tandem repeat or a microsatellite repeat
